# A relational approach to consciousness: categories of level and contents of consciousness

**DOI:** 10.1093/nc/niab034

**Published:** 2021-10-15

**Authors:** Naotsugu Tsuchiya, Hayato Saigo

**Affiliations:** School of Psychological Sciences and Turner Institute for Brain and Mental Health, Monash University, Melbourne, VIC 3800, Australia; Center for Information and Neural Networks (CiNet), National Institute of Information and Communications Technology (NICT), Suita, Osaka 565-0871, Japan; Advanced Telecommunications Research Computational Neuroscience Laboratories, 2-2-2 Hikaridai, Seika-cho, Soraku-gun, Kyoto 619-0288, Japan; Nagahama Institute of Bio-Science and Technology, 1266 Tamura-cho, Nagahama, Shiga 526-0829, Japan

**Keywords:** consciousness, contents of consciousness, level of consciousness, qualia, category theory, yoneda lemma, functor, natural transformation

## Abstract

Characterizing consciousness in and of itself is notoriously difficult. Here, we propose an alternative approach to characterize, and eventually define, consciousness through exhaustive descriptions of consciousness’ relationships to all other consciousness. This approach is founded in category theory. Indeed, category theory can prove that two objects A and B in a category can be equivalent if and only if all the relationships that A holds with others in the category are the same as those of B; this proof is called the Yoneda lemma. To introduce the Yoneda lemma, we gradually introduce key concepts of category theory to consciousness researchers. Along the way, we propose several possible definitions of categories of consciousness, both in terms of level and contents, through the usage of simple examples. We propose to use the categorical structure of consciousness as a gold standard to formalize empirical research (e.g. color qualia structure at fovea and periphery) and, especially, the empirical testing of theories of consciousness.

## Problems in characterizing consciousness on its own

Over the last few decades, the question of the nature of consciousness is gaining a respected position as the target of scientific inquiry. Much of the empirical research has tried to identify the neural correlates of consciousness (Koch *et al.* 2016). Building on the massive amount of empirical evidence, some models or theories have been proposed to explain the link between various features and aspects of the neural activity and the associated functions and phenomena of consciousness (Engel and Singer 2001; Graziano and Kastner 2011; Lamme 2015; Northoff and Huang 2017; Brown *et al.* 2019; Mashour *et al.* 2020).

Another distinct approach to consciousness is to study it using a mathematical framework (Hoffman 1966, 1980; Stanley 1999; Tononi 2004; Fekete and Edelman 2011; Yoshimi 2011; Prentner 2019; Kleiner 2020; Prakash *et al.* 2020; Signorelli *et al.* 2021). Among these, the Integrated Information Theory (IIT) of consciousness by Tononi and colleagues (Oizumi *et al.* 2014; Tononi *et al.* 2016) is arguably the most developed and discussed in the literature. IIT takes a unique approach, where it tries to first identify the essential properties of consciousness, which are always true to any experience. These essential properties are called “phenomenological axioms,” and IIT tries to derive mathematical postulates that any physical system should satisfy to support these properties. IIT then proposes an explanatory “identity” between phenomenal consciousness and information structure (Haun and Tononi 2019).

In the past (Tsuchiya *et al.* 2016), we, the authors, have suggested that, rather than trying to propose the explanatory “identity” between the two in a single step, it would be empirically more tractable to break up the IIT project into the following three subprojects: (i) to characterize the structure of conscious phenomenology as a category; (ii) to characterize the structure of information as a category; and (iii) to assess the degree of similarity between the structures. For the second issue, we and others have made some initial efforts on the mathematical side (Northoff *et al.* 2019; Kleiner and Tull 2020; Tull and Kleiner 2020). Notably, there has been progress on the application of category theoretical approaches toward stochastic processes (Manin and Marcolli 2020) to formalize information structures (Baudot and Bennequin 2015; Baudot *et al.* 2019). For the third issue, we also made an initial attempt (Haun *et al.* 2017). Here, we deal with the first issue: how we can characterize the structure of consciousness as a category. Note that category theory has been proposed to model aspects of cognition and how they are supported by neural networks in the brain (Ehresmann and Vanbremeersch 1987, 1996; Arzi-Gonczarowski 1999; Healy *et al.* 2008; Phillips and Wilson 2010, 2016; Ehresmann and Gomez-Ramirez 2015; Phillips 2020).

Precisely defining consciousness has been notoriously difficult (Dennett 1988; Sloman 1991; Stanovich 1991; Chalmers 1996; Velmans 2009; Kleiner 2020), but certain characterizations of consciousness are accepted by many for use in empirical consciousness research (Koch *et al.* 2016; Mashour *et al.* 2020). One of the widespread consensus is to distinguish the “level” and “contents” of consciousness (Laureys 2005; Boly *et al.* 2013). For example, Searle (Searle 2000) gives the following definition: “Consciousness consists of inner, qualitative, subjective states and processes of sentience or awareness.” Consciousness, so defined, begins when we wake in the morning from a dreamless sleep and continues until we fall asleep again, die, go into a coma, or otherwise become “unconscious.” In the case of the contents of consciousness, a particular conscious experience of red color, often called a red quale (Kanai and Tsuchiya 2012), is typically characterized as “redness of red” or “red like this wine.” In these cases, consciousness is characterized in reference to another conscious experience. This relational nature of consciousness appears to be one of the fundamental characteristics of consciousness (Nagel 1974; Chalmers 1996; Kleiner 2020).

In this paper, by introducing several notions of category theory, we offer mathematical justification for the relational characterization of consciousness. In fact, situations where a particular object is not possible to define or even characterize on its own arise relatively often outside of consciousness research. In these situations, what has been effective is to rely on the relationships between the object to be defined and its surroundings. For example, some linguists consider that meanings of a word can be understood only through how the word is related to other words and how they are put into the context in the sentence (e.g. Frege 1980; De Saussure 2011). In ecology, it is essential to characterize any life form within an ecosystem; defining a tree without mentioning the geological area in which it lives and which animals, insects, and other plants interact with it and in what way would miss the very essence of what that tree is. In quantum theory, the essence of quantum features is that they are only possible to explain through the interactions between the objects (Coecke and Kissinger 2017). In cosmology, black holes are, in themselves, impossible to measure and characterize, but their interaction with their neighbors can be measured and used to characterize their properties. In mathematics, various types of infinity can be distinguished through what types of relationships they have with other mathematical objects.

There is a deep mathematical foundation in why these theoretical fields can dispense with a direct definition of an object in favor of characterizing its interaction with its surroundings. That is what we introduce to consciousness research in this paper: the Yoneda lemma. In short, the Yoneda lemma allows us to equate A with B (up to isomorphism defined in “What does it mean for objects to be the same in a category? Isomorphic objects in a category”) if the relationships between A and the rest of objects (including B) are the same (up to natural equivalence defined in “Functor category, whose objects and arrows are functors and natural transformations”) as those between B and the rest (including A). Importantly, this is true, even if A and B themselves are difficult to characterize in and of themselves, as in the case of black holes in cosmology or infinity in mathematics. While defining consciousness directly in a way where everyone agrees is not easy, characterizing consciousness through a rich set of relationships is much more feasible.

Note that we will not offer a definitive definition(s) of consciousness in this article. Rather, our plan is to introduce a novel perspective on how we can start doing so through the application of the Yoneda lemma. Through our proposed approach, eventually, we would hope to completely justify the relational definition of consciousness. But first things first. To apply the Yoneda lemma, we need to propose several possible categories of consciousness. After introducing key concepts in category theory, we will come back to the issue of how we can apply the Yoneda lemma in consciousness research and discuss what it means for consciousness research. For example, the Yoneda lemma will enable us to address the question of the equivalence of color qualia structure at the fovea and the periphery in a systematic way.^1^

## What’s the category in category theory?

### Categories of consciousness

In this section, we will explain the basics of category theory, which is necessary to explain the Yoneda lemma with a view to its application in consciousness research. We will propose two categories: category of level of consciousness, Lv, and category of contents of consciousness, Q Note that we are not claiming that these examples are “the” only possible formulation of categories of consciousness. They are invoked here as a starting point. For an accessible introduction to category theory, see Lawvere and Schanuel (2009); Spivak (2014); Bradley (2017); Northoff *et al.* (2019).

The basis of category theory is three concepts: (i) category, (ii) functor, and (iii) natural transformation. Let us start with a category.

**Definition:**  *For a collection of objects to be considered as a category, they must satisfy the following*  *five axioms.*

1. *An arrow has its “* *source”*  *object called domain and “target”*  *object called codomain.*
2. *For every object, there is a self-referential arrow called identity.*
3. *A pair of arrows is*  *composable if the domain of one arrow equals the codomain of another.*
4. *Identities do not change other arrows by composition.*
5. *Composition is associative.*

**Figure 1.**
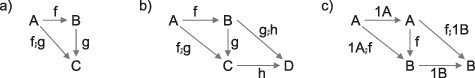
Requirements of a category. (a) Composition: if A, B, and C are objects in category X, and f: A → B and g: B → C are arrows in category X, then we can compose (or combine) f and g to obtain an arrow, f;g: A → C. Note f;g reads as “f then g” and it is often denoted as g°f (Fong and Spivak 2019). (b) Associativity: if f, g, and h are arrows in category X, then the order to compose the arrows does not matter: (f;g);h = f;(g;h). A, B, C, and D are objects in category X. (c) Unit: For any object A in category X, there is a self-referential arrow A → A, which is called identity arrow: 1A. For any arrow f: A → B, the following is always satisfied: 1A;f = f = f;1B

In other words, a category is a system consisting of “objects” and “arrows.” Figure 1 explains the above definition with a diagram in an intuitive manner.

For a category of level of consciousness, we consider global states of consciousness or the degree of wakefulness as an object. The level of consciousness is usually assumed to go from 0 in dead humans, which is lower than deeply anesthetized or dreamless sleep, and highest in fully wakeful states (Mormann and Koch 2007). Note that one object can contain many elements. As an example, let us consider a fully wakeful state as an object. One can distinguish each moment of experience as different elements. An object in this case is a group of these elements considered as one entity, much like the colloquial use of a “category.” Mathematically speaking, an object does not have to be one element.

For a category of contents, we can consider any possible content of consciousness, such as color, sound, and pain. As is usually the case in mathematics, however, starting from the most general situation, encompassing all levels of consciousness in all animals or all types of contents, is not a wise strategy to make progress. Accordingly, we will consider a very concrete case of consciousness categories below and leave generalization of these categories to future work.

An arrow, →, relates an object with another object. For example, f: A → B denotes a relationship f between object A and object B. In the case of level of consciousness category Lv, we can define the meaning of f to be the level of consciousness in A “is higher or equal to” B. Soon, it will become clear why we need “equal to.” Read on! In the case of content category Q, we can define the meaning of f to be that A “is experienced as nearly indistinguishable with” B in a certain aspect.

If the system consisting of objects and arrows satisfy further three conditions (composition, associativity, and unit) as depicted in Fig. 1, then it qualifies as a category. We visualize these three requirements concisely and intuitively using diagrams. This is one of the most powerful aspects of category theory: simply visualizing complex relationships to facilitate the understanding of the subject matter.

### A category of level of consciousness

Let us verify whether our proposed objects and arrows for level of consciousness, Lv, can qualify as a category. Objects are certain levels of consciousness supported by a human brain, such as A, B, C, etc. An arrow f exists between two conscious levels A and B, if level of consciousness A “is higher than or equal to” B.

Consider the requirement of composition. Suppose that f: A → B and g: B → C exist as arrows for objects A, B, and C in consciousness level category Lv. That is, the level of consciousness A is higher or the same as B, and B is higher or the same as C. Then, it follows that A is higher than C or the same. In other words, there is an arrow from A to C, which is the combination of f and g, which we denote as f;g. Therefore, the condition for composition is satisfied.

Next, let us consider associativity. We assume that for levels of consciousness A, B, C, and D, three arrows exist f: A → B, g: B → C, and h: C → D. Then, (f;g);h means that (i) we first confirm that A is higher than or the same as C (i.e. f;g) and (ii) due to h (i.e. C is higher than or equal to D), we conclude that A is higher than or the same as D. On the other hand, f;(g;h) means that (i) we first confirm g;h, that is, B is higher than or equal to D, and (ii) we conclude A is higher than or equal to D. In other words, associativity requires these two conclusions to be exactly the same. In the diagram (Fig. 1b), this translates to the fact that two conclusions via two paths are exactly the same if the starting and the ending points are the same.

Finally, let us check the unit. This is the reason why we have added “or equal to” as a part of the definition of the arrow. For any level of consciousness A, there exists an arrow 1A: A → A because A is always higher than or equal to A. If f:A → B exists, then If f: A → B exists, then 1A;f means the following. (i) We confirm that A is the same as A. Then (ii) we conclude that A is higher than or equal to B. Similarly, f;1B means the following. (i) We confirm A is higher than or equal to B. Then, (ii) we confirm B is equal to B. If we define f: A → B as A is strictly higher than B, our proposed system of objects and arrows for level of consciousness Lv does not qualify as a category.

Our category of level of consciousness Lv is an example of categories called “preordered sets.” Preordered sets are categories such that between any two objects there is at most one arrow. The arrows in preordered sets are called “preorder.”

### A category of contents

Next, let us examine if our proposed definition can make a system of contents of consciousness Q into a category. Here, objects are the contents of consciousness. Let us consider a system Q that consists of only three objects, A = red sunset, B = red crayon, and C = red wine. For simplicity, we consider a case of a preordered set; that is, between two objects, there is either one arrow or none at all. Suppose there is an arrow from A to B when the contents of the two consciousness are “nearly indistinguishable” in terms of their color [for those who are concerned about the gradual degradation of distinguishability, see Tsuchiya *et al.* (2021)]. In other words, if there is an arrow f between the two contents of consciousness A and B, then f: A → B, and A is subjectively felt as nearly the same as B in terms of their color. It is clear that the composition holds. In other words, if the redness of sunset and crayon are similar, and the redness of crayon and wine are similar, then the redness of sunset and wine are also similar. Mathematically speaking, if f: A → B and g: B → C, then f;g: A → C also holds. Associativity holds as well. The unit is also valid (these two proofs are simple and left to the reader). Therefore, our proposed system of objects and arrows Q can constitute a category.

Here, since arrows mean “nearly indistinguishable,” the direction of an arrow does not matter. Now that we are considering a preorder, there is only one arrow in either direction. This implies that any arrow in this category is invertible. That is, any arrow has an arrow of a reverse direction such that the composition of the two is the identity arrow. Invertible arrows are also called “isomorphism” not only in preordered sets but also in categories in general. When all arrows are isomorphism, the category is called “groupoid.” In sum, we proposed an exemplar category Q, which is preorder and groupoid, where all arrows are isomorphism (if there are multiple isomorphisms between objects, then such a category is groupoid but not preorder).

### What does it mean for objects to be the same in a category? Isomorphic objects in a category

So far, we have introduced categories Lv and Q for level and contents of consciousness. Next, to deepen our understanding of what it means to define an object, we will consider what it means for objects A and B to be equivalent.

When we say that A and B are the “same” in everyday life, it means that they are equivalent with respect to some definitions or assumptions and ignoring various aspects. For example, when there are two apples on the right and the left, they may be similar in terms of their color or a category within the fruit, but they are different in their surface textures. In fact, they are not the same thing, being located in different places. Category theory provides a wealth of mathematical tools to handle these subtle differences in “sameness” (Tsuchiya *et al.* 2016) in that it characterizes different kinds of sameness using various types of relationships.

Let us explain the sameness between objects in a category, called “isomorphism.”

**Definition:**  *The objects A and B in a certain category C are isomorphic if there is an “invertible”*  *arrow (called isomorphism) between them. When the arrow f: A*  *→*  *B is “invertible,”*  *there exists an arrow g: B*  *→*  *A with f*;*g = 1A and g*;*f = 1B.*

In the case of category Lv, if coma level A is lower than minimally consciousness level B, the reverse of the arrow f: A → B does not exist. In our proposed example of the category of contents with three objects, all arrows are isomorphisms (see Section 2.3), that is, any pair of objects is isomorphic if there exists an arrow between them.

This concept of isomorphism is much looser and more flexible than the usual concept of “equalities.” For example, under the framework of set theory, a set of alphabets {a, b, c, d, e} is not equal to a set of numbers {1, 2, 3, 4, 5}. The difference in terms of “elements” disqualifies them to be considered as equal sets. Now, consider a category Set whose objects are sets and arrows are functions or mappings between sets. Within the category Set, two objects (i.e. sets) {a, b, c, d, e} and {1, 2, 3, 4, 5} are considered as isomorphic objects in the sense that each element can be mapped from one set to the other set in a one-to-one manner. In fact, a concept of a number, such as 5, is “defined” when we regard these “different” sets as the “equivalent” in a certain respect. In this sense, isomorphism captures the concept of “essential sameness” in the category. As another example of isomorphism between objects in a category, consider a category of topological space, Top, which is the foundation of the mathematical field called topology. In Top, objects are topological spaces and arrows are continuous maps. While coffee cups and donuts are usually considered as “completely different” objects, they are isomorphic in Top.

In order to make progress in consciousness research, equivalence in the sense of isomorphism is not powerful enough. As we discuss later, we believe that different types of sameness in category theory will be more useful and find wider application in consciousness research. In particular, isomorphism between categories, or even looser sameness, called categorical equivalence will be critical. To introduce these concepts, we need to introduce two concepts: functors and natural transformations, which are the topics of the next two chapters.

### Preliminary conclusion and discussion

We believe that introducing the concept of preordered sets as a category of level of consciousness Lv has an important implication in consciousness research. Some authors have raised issues with the concept of level of consciousness (Bayne *et al.* 2016; Pautz 2019). One of the issues is that this concept seems to imply a gradual change from unconsciousness to full consciousness, which is not widely agreed upon. Another issue is the existence of certain pairs of conscious states, where we can never subjectively compare which state is “higher” as conscious level. For example, which is higher in level of consciousness between vivid dreaming and drowsy awakening states? Or, what about between deep general anesthesia and coma, where consciousness completely disappears? We believe that these issues arise because the scholars who criticize the concept of level of consciousness implicitly assume that “level” is something that is “isomorphic” to natural numbers (or positive real numbers), where it is always possible to rank the order between two objects. Such a strongly ordered structure is called “total order” in mathematics. We believe that the total order assumption is not necessary at all for the concept of level of consciousness to be useful. We propose that a looser concept, preorder, is a useful and appropriate concept for level of consciousness. As for preorder, any two objects (levels) may or may not have a relationship “≦.”

Our proposed category Lv can be applied to many cases. Compared to the fully wakeful states, deeply anesthetized, dreamless sleep, or coma would be lower in the level of consciousness. In Lv, we will have no arrows between cases of lower level of consciousness, where subjects have no ability to compare their levels and do not show any behavioral outputs to be meaningfully compared. Likewise, Lv can be proposed across animals without proposing any arrows between animal species but only considering arrows within animals (between fully wakeful and deeply anesthetized, deep sleep, or coma).

Note that the two categories Lv and Q proposed here are just toy examples and we are not claiming that they are “the” categories of consciousness. In both level and contents of consciousness, it is possible to focus on other aspects of consciousness and consider the corresponding categories. For example, by focusing on an aspect of consciousness that changes over time, one can propose a “category of mobility” (Saigo *et al.* 2019). Another kind of category, called “co-slice category,” is effective in capturing association possibilities and metaphorical structures of meaning (Fuyama and Saigo 2018) [note that the subjective experience of “meaning” of a word is not accepted widely, however; see Kemmerer (2015) and McClelland and Bayne (2016) for recent discussions on this issue]. Tsuchiya *et al.* (2021) explains a further framework to loosen the condition of arrows in Q by making it in variable degrees of similarity using a concept of enriched categories (Lawvere 1973; Leinster and Meckes 2017; Fong and Spivak 2019).

The point of this section was to provide the readers an understanding that it is not difficult to propose a category of consciousness. Some readers may think that as a mathematical theory, category theory cannot be applied to the problem of consciousness, especially because of the problem in composition (Pitt 2018). As we explained, however, category theory can be applied if “objects” and “arrows” are appropriately defined and if they satisfy a few conditions.

## What is a functor?

### A functor is an arrow between categories

**Figure 2.**
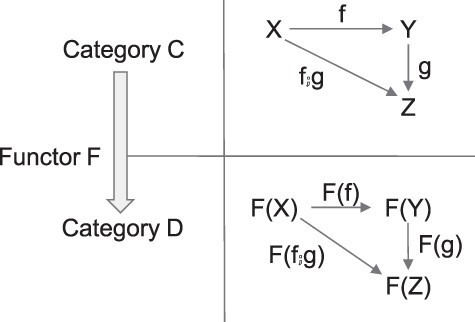
What is a functor? A structural mapping F between category C to D is called a functor if the following three conditions are satisfied: (i) an arrow f: X → Y in category C is mapped onto a corresponding arrow F(f): F(X) → F(Y) in category D; and (ii) a composite arrow f;g in category C is preserved as F(f;g) = F(f);F(g); and (iii) an identify arrow 1X for an object X in category C is preserved as F(1X) = 1 F(X)

In simple terms, a functor is an arrow between two categories (for a graphical definition, see Fig. 2). Importantly, a functor needs to map one category to another category while keeping the “structure” of the category (e.g. commutativity). From the viewpoint of equivalence discussed in the last chapter, “the existence of a functor” is one important condition to consider as the sameness between categories. The sameness as “existence of a functor” is much weaker than the other types of sameness discussed in this paper.

**Definition:** *A mapping F, which maps an object and an arrow in category C to an object and an arrow in category D, is called a functor if it satisfies the following three conditions:*

1. *F(1X) = 1 F(X), that is, the identity arrow of X will map to the identity arrow of F(X).*
2. *f:X → Y will map to F(f):F(X) → F(Y), that is, the arrow from X to Y maps to the arrow from F(X) to F(Y).*
3. *a composite arrow f;g in category C is preserved as F(f;g) = F(f);F(g).*

To see the relevance of functors in consciousness research, let us examine a functor from categories of level of consciousness Lv. To remind you, Lv is a preordered set; objects and arrows in Lv are level of consciousness and “≦” as introduced previously. As we argued ‘there’ the construction of category Lv and clarifying its specific structure itself is a difficult research program in itself. But here, we focus on the other problem: to establish a consciousness meter, which translates to a construction of a functor from category Lv to a convenient preorder category D. As an example, consider a category D whose objects and arrows are natural numbers and ≦. Readers can check easily that D is a preorder category; any pair of objects have at most one arrow (e.g. 1 ≦ 2), any arrows can be composed in an associative manner (e.g. 1 ≦ 2 ≦ 3) and that each object has a unit arrow (e.g. 1 ≦ 1).

Let us consider a mapping F that maps from category Lv to category D and satisfies the conditions to be a functor. In other words, F maps any object of Lv, that is, any level of consciousness, into a natural number, while all ≦ relations in Lv are preserved in D. In other words, searching for a functor F is the same as the construction of a consciousness meter as strived for by some consciousness researchers. We also note here that mapping in the other direction, that is, from D to Lv does not have to be a functor. In other words, the validity of a consciousness meter, F, is not compromised if two objects with one arrow (e.g. 1 ≦ 2 in D) do not map onto Lv functorially. For example, from D to Lv, a mapping G may map 1 to coma and 2 to deep general anesthesia. If G does not map ≦ in D into ≦ in Lv, then G is not a functor. However, as we pointed out in the last section, it is unclear whether ≦ exists between coma and deep anesthesia; thus, this itself is not a problem for F as a valid consciousness meter.

**Figure 3.**
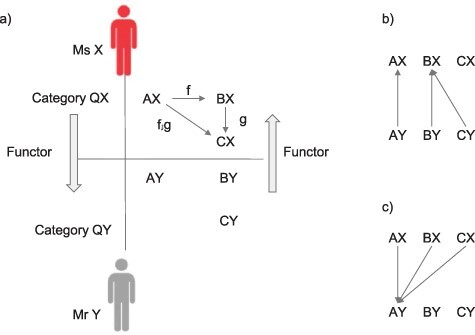
Exemplar functors between categories QX and QY. (a) In category QX, there exist arrows for all possible object pairs (only three arrows are depicted here). In category QY, there are no arrows except for identity arrows for each object (not drawn). (b) One of 27 (=3^3^) functors from QY to QX that preserve structures of QY. (c) One of three functors from QX to QY. This functor has to map all objects in QX into one object in QY and all arrows in QX to the identity arrow in QY

Next, let us consider a functor F for category Q for contents of consciousness as considered in the last section. Q is a groupoid, and its three objects are A = red sunset, B = red crayons, and C = red wine. Arrows are “nearly indistinguishable” in terms of color. Here, we consider two persons’ categories QX and QY. For Ms X, all objects’ colors are nearly indistinguishable; thus, there are arrows for any pair of objects AX, BX, and CX. However, for Mr Y, all objects are distinguishable; thus, there are no arrows among AY, BY, and CY (Fig. 3a).

In this situation, there are 27 functors that map from category QY to category QX since the destination of each object in category QY can be any of AX, BX, and CX. For example, one functor F1 maps AY to AX, while it maps BY and CY into BX (Fig. 3b). The only arrows in QY are the identity arrows. The identity for AY maps to that for AX, while those for BY and CY maps to that for BX. The requirement of composition is also satisfied (because the only way to compose arrows in QX is to compose the identity with the identity, which is the identity).

What about functors from category QX to category QY? Like the above example, if F collapses all objects in QX into one object in QY, F can satisfy all conditions to be a functor. If F does not collapse them, what happens? For example, if AX is mapped to AY, while BX is mapped to BY. Because Mr Y can distinguish AY and BY, there is no arrow between AY and BY. However, Ms X sees AX as indistinguishable from BX. Thus, the arrow that connects AX and BX is lost in translation during this mapping. Such a faulty mapping does not qualify as a functor. From a viewpoint of equivalence, there is a certain “structural similarity” in subjective similarity characterized by categories QX and QY in a sense that there exists a functor from one category to the other. But the richness of the structure is “different” as can be quantified from the number of arrows in each category. This nicely represents the situation of the subjective experience of Ms X and Mr Y—they experience the objects with certain structural similarity and difference in richness in the structure.

In sum, functors can be used to compare structures between categories. In terms of categories of contents of consciousness Q, functors are not limited in the usage of comparison of similarity structures among individuals as in our examples. Functors can be used to compare structures of content category of color with content category of shape, content category of meaning, etc. within individuals. This is one of the possible future directions of category theoretical research of conscious contents.

### Category of categories

In a category of categories, Cat, objects and arrows are categories and functors (and this is the origin of the title for the last section). This is an interesting characteristic of category theory; depending on the viewpoint, what we are talking about can be considered as objects in a certain category or arrows in another category. Furthermore, a functor, which is considered as an arrow between two categories in this section, will be considered as an object in the later section. All of this comes from the fact that category theory is mathematics that focuses on “relationships.”

Let us consider a category of categories using an example of category of content Q. We ask Ms X and Mr Y to come back on the stage. If we consider category QX and QY as objects and functor F as arrows, the example in Fig. 3 is an exemplar category of category Q.

## What is a natural transformation?

A natural transformation is the central concept in category theory. Indeed, category theory was originally invented to introduce the concept of natural transformation. To explain natural transformation, the concept of functor was introduced. And to explain the concept of functor, the concept of category was introduced. Natural transformations are necessary to state and prove the Yoneda lemma. The Yoneda lemma and its inspiration for consciousness research is the central message of the paper: even if it is difficult or impossible to characterize consciousness per se, we can do so by characterizing all arrows in the relevant category.

### A natural transformation is an arrow between functors

**Figure 4.**
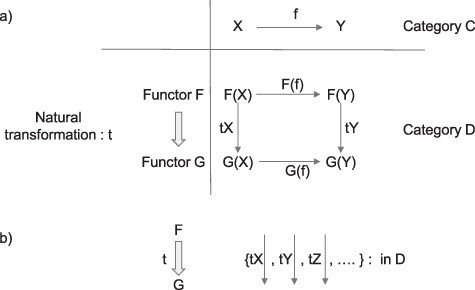
What is a natural transformation? (a) Category C (consisting of objects X and Y and an arrow f) is mapped into category D by two functors F and G. Each functor preserves the structure of category C in D (e.g. F(f):F(X) → F(Y), G(f):G(X) → G(Y)). Natural transformation t maps functor F into G while preserving its structure. t can be considered as a family of arrows in category D, such as tX and tY. Each arrow is specified by an object in the original category C, such as tX, which maps an object F(X) to G(X) in category D. b) Visualizing t as a family of arrows in the destination category D

A natural transformation maps a functor to another functor while keeping the structure of the functor. The basic structure of category theory can be stated in terms of categories, functors, and natural transformations. While there are more, solid understanding up to natural transformation is critical to handle category theory. It is important to develop an intuition about a natural transformation. It is a family of arrows in the category, which a functor maps the original category into. The graphical definition in Fig. 4 helps understand this concept.

**Definition:**  *Consider functors F and G that map category C to category D. When a mapping t from functor F to G satisfies the following two conditions, t is called a natural transformation and we write t: F*  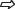  *G.*

1. *Given an object X in category C, t gives an arrow tX: F(X) → G(X) in category D.*
2. *For any arrow f: X → Y in category C, tX*;*G(f) = F(f)*;*tY holds in category D.*

*tX is called the X component of t.*

#### Natural transformation for category Lv

To understand the relevance of natural transformations in consciousness research, let us first consider the category of level of consciousness Lv and the functors. Suppose Ms F proposed a functor F as a consciousness meter, which maps all levels in preorder category Lv to preorder category D (objects and arrows are natural numbers and ≦). F maps all objects in Lv to numbers in D and ≦ in Lv into ≦ in D. On F’s monitor, level of consciousness under deep general anesthesia shows up as number 10, while that under the wakeful state is 100. Then Mr G also developed another functor G as a consciousness meter. On G’s meter, the level under deep general anesthesia is 20 and the wakeful state is 200. In both cases, the arrow in category C is preserved as the arrow in category D as ≦. Now, we ask: what is the nature of the relationship between F’s and G’s consciousness meters?

Let us assume that there exists a natural transformation t between functors F and G. Then, according to Condition 1 above, the natural transformation t gives us two arrows tA and tW for two objects that we considered in category Lv: A for anesthesia and W for wakefulness. tA and tW are two arrows in category D, which satisfies the following: tA: F(A) = 10 → G(A) = 20, tW: F(W) = 100 → G(W) = 200. Interestingly, this arrow corresponds to ≦, the arrow in D.

Moreover, according to Condition 2, with respect to two objects A and W and the arrow f:A → W in category Lv, we have tA;G(≦) = F(≦);tW. The left-hand side of this equation means that (i) we first evaluate the value for anesthesia in F (=10) to translate into the value in G (=20), then (ii) we confirm the relation between A and W (≦) in category Lv to be translated into the relation in G’s monitor (20 ≦ 200). The right hand side of the equation means that (i) we first translate the relation A ≦ W in category Lv into category D (F(A) = 10 ≦ F(W) = 100) and then (ii) we map the value for wakefulness in F’s monitor (=100) into G’s monitor (=200). In sum, this family of arrows in category D guarantees a lawful relationship between two functors, preserving the original relations in category Lv (note that we also have two more relations with respect to the identity arrows for 1A and 1 W in category Lv).

#### Natural transformation for category Q

**Figure 5.**
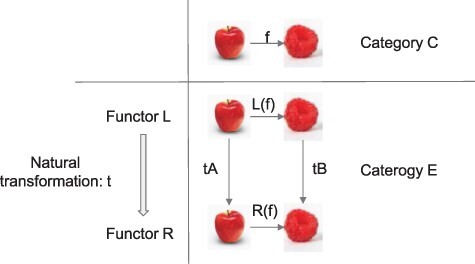
Natural transformation. Objects are Apples (A) and Berries (B) in categories C (central visual field) and E (entire visual field). Arrows in categories C and E are “nearly indistinguishable” in terms of color. Thus, category C is included in E. Central vision (C) is mapped to Left and Right visual fields by functor L and R, respectively, preserving objects and arrows. If natural transformation t exists from functor L to functor R, then indistinguishability of Left Apple to Right Berry can be established by both through Left Berry (=L(f);tB) or Right Apple (=tA;R(f)), that is, there is no path dependency. Note that tA is an arrow in category E, indicating that Apple in the Left is “nearly indistinguishable” from Apple in the Right. Same goes with tB for Berry in the Left and Right

Next, let us consider a natural transformation for the category of contents Q. We consider two categories based on Q introduced in the section on “A category of contents” and consider a situation that can be tested in a psychophysical experiment. For simplicity, we consider two objects as subjective experience of a red Apple (A) and a red Berry (B) and arrows as a relationship for “nearly indistinguishable” in terms of color (Fig. 5). Category C refers to objects and arrows in the Central visual field, while category E refers to those in the Entire visual field, which includes the central, left, and right visual field. A mapping R from C to E translates objects and arrows to those in the Right visual field. Likewise, a mapping L from C to E translates those in the Left visual field. The mappings L and R are likely to be functors in healthy subjects. One way to test this idea is to operationally define category C with objects as a set of perceptual objects and arrows as similarity relationships. Likewise category E, which includes C but also across the entire visual field. Under this condition, psychophysical experiments measuring similarity relationships can test if the mapping L and R satisfies the functor conditions. In the following, we assume L and R satisfy the functor conditions.

Natural transformation t can clarify the relationship between functors L and R. Let us go through this example slowly as this is an important point. Assume the existence of a natural transformation t from functor L to functor R. Now, with respect to objects A (Apple) and B (Berry) as well as its arrow f (f:A → B; A is nearly indistinguishable from B in terms of color) in category C for Central visual field, a natural transformation is a collection of arrows in category E for Entire visual field. These arrows include tA: L(A) → R(A) and tB: L(B) → R(B) (Condition 1), both of which mean “nearly indistinguishable” in terms of color, which are indeed the arrows in category E. tA means that Apple is nearly indistinguishable in terms of color in the Left and Right visual field.

Condition 2 says for an arrow f:A → B in category C, we have tA;R(f) = L(f);tB. The left side of this equation first confirms that Apple is indistinguishable between Left and Right visual fields, then it confirms that Apple in Right is indistinguishable from Berry in Right. The right side of the equation first confirms that Apple is indistinguishable from Berry in Left, then Berry in Left is indistinguishable from Berry in Right. In other words, Condition 2 means that the color of Left Apple is indistinguishable from the color of Right Berry, and it allows us to go in either way to prove this fact. The power of the natural transformation t is to handle a lot of arrows, such as tA and tB, all at once in a lawful manner.

### Functor category, whose objects and arrows are functors and natural transformations

Using the concept of natural transformation, we introduce another critical concept to prove the Yoneda lemma: functor category. Functor categories and equivalence defined in this manner, we suspect, is something that is likely to be completely missing in current language in consciousness research, and it is very useful for future conceptual analyses.

**Definition:**  *Functor category, Fun(C, D), considers objects and arrows as functors from category C to category D and natural transformations between them*.

From the viewpoint of the contents category, let us consider a functor category. We extend the example categories C and E in Fig. 5. Simply, treating functor L and R as objects and a natural transformation t as an arrow constructs a functor category Fun(C, E).

What is the benefit of considering a functor category? One of the benefits is to allow us to introduce two concepts: “natural equivalence” and “categorical equivalence.” Categorical equivalence is likely to be a critical target to establish in empirical studies of the structure of consciousness within a framework of category theory; for example, one application is the examination of categorical equivalence of color similarity structure at the fovea and the periphery.

Previously, peripheral and central color vision have been claimed to be “essentially the same” (Tyler 2015; Haun *et al.* 2017) or not (Dennett 1991; Lau and Rosenthal 2011). Between these visual fields, objective resolutions are different; yet, when the size of objects are matched for the resolution, essential visual phenomenology, including color experience, seems “essentially the same” (Gordon and Abramov 1977; Hansen *et al.* 2009; Webster *et al.* 2010). How can we proceed to extract and quantify the sameness of obviously different objects?

First, you might think of “isomorphism” between category C and E for Central and Entire visual fields, respectively (Remember the section on “What does it mean for objects to be the same in a category? Isomorphic objects in a category”). As we introduced in the section “Category of categories”, C and E are objects of Cat (category of categories), where arrows are functors. Isomorphism in Cat, which is also called “categorical isomorphism,” requires that, for functor F: C → E, there exists functor G: E → C such that 1 C = F;G and 1E = G;F. This turns out to be a very strong requirement and it does not work in the case between C and E here. This is because functor G collapses E into its subset C; thus, F cannot recover the original E from C.

On the other hand, what occurs if we consider the “isomorphism” instead of equality in 1 C = F;G and in 1E = G;F? “Isomorphism” here is nothing but an invertible natural transformation, which is called natural equivalence, in functor categories Fun(C, C) and Fun (E, E). In this case, we obtain a weaker, yet very proper and flexible, kind of equivalence. In this sense, two categories C and E are equivalent. This is the level of equivalence that we are looking for, and potentially useful for consciousness research. The sameness, obtained through natural equivalence, is called “categorical equivalence” between category C and E.

**Definition:**  *Categories*  *C and E are categorical isomorphic if there are functor F: C*  *→ E and functor G: E → C*  *such that 1 C = F*;*G and 1E = G*;*F. Category C and E are categorically equivalent if there are invertible natural transformations from 1C to F;G and 1E to G;F*

In our example of Central and Entire visual fields (Fig. 5), C and E will not be “categorically isomorphic,” which implies one-to-one relation on object and arrows since a functor from E to C should collapse the multiple objects or arrows into the same object or arrow. On the other hand, C and E can be “categorically equivalent” since it allows such multiplicity up to isomorphisms. If this can be experimentally verified, then we can conclude that content structures are “essentially the same” across visual fields. If, however, something essential is lost within a part of the visual field, say, blindspot, scotoma (Ramachandran and Blakeslee 1998), or colorblindness in a quadrant (Gallant *et al.* 2000), then, categorical equivalence would not hold any more.

Empirically, we can possibly test the existence of categorical equivalence between color similarity structures as content categories between the fovea and the periphery. While color similarity structure has been extensively studied in psychophysics (Kuehni 2010), most experiments allowed subjects to move their eyes freely in an unlimited time. As a result, the structure of color experiences is understood better at the fovea than the periphery, resulting in disputes (Cohen *et al.* 2020; Cohen and Rubenstein 2020; Dennett 1991; Lau and Rosenthal 2011; Tyler 2015; Haun *et al.* 2017). We surmise that a weaker notion of categorical equivalence is much more likely to be applicable in various types of structures of consciousness and more fruitful to seek for than the other stronger notions of the sameness (e.g. categorical isomorphism).

## Explaining the Yoneda lemma

Finally, we are ready to introduce one of the most important results of category theory, the Yoneda lemma, to consciousness research. If we can apply the Yoneda lemma to consciousness research, we can characterize consciousness through its relationships with the others even if we cannot describe what consciousness is per se. To be more precise, the general conclusion of the Yoneda lemma is that the characterization of an object in a category is determined up to isomorphism by its arrows to the other objects in that category. We believe this is a substantial change of perspective, especially in the context of consciousness research: properties of an object are essentially the same as how the object relates with the others.

Having said that, some may say that a concise and general direct definition of an object is vastly superior to the exhaustive characterizations of its relationships to other objects. However, as we noted in problems in characterizing consciousness on its own, the difficulty in dealing with consciousness is its difficulty to describe it in itself. How can I examine my definition of “redness” at the fovea compared to my “redness” at the periphery? As a start, I can compare my “redness” at fovea to all other experiences, including “redness” at periphery. Next, I can do the same by comparing my “redness” at periphery to all other experiences. This is an empirical approach to characterize a structural relationship. This procedure can establish equivalence of color experiences at the fovea and the periphery. The Yoneda lemma provides a mathematical footing to this empirical approach: we can eventually establish equivalence of my redness across the visual fields.

### Hom sets and hom functors

Before applying the Yoneda lemma to consciousness categories, let us introduce only two more concepts: hom sets and hom functors.

**Figure 6.**
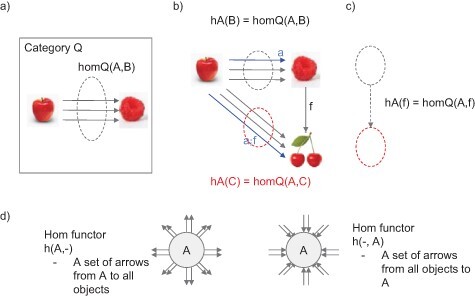
What are hom sets and hom functors? (a) A hom set, homQ (A, B), is a set of arrows from Apple (A) to Berry (B) in category Q. homQ (A, B) includes our familiar arrow, “nearly indistinguishable in color” but also many other different arrows (e.g. “indistinguishably tasty,” “indistinguishably sour,” etc). (b) A hom functor, hA, maps objects B and C in category Q into objects hA(B) and hA(C) in category Set. (c) hA also maps an arrow f in category Q into an arrow in category Set. (d) Visual depiction of hA: all sets of arrows emanating from A; and Ah: all sets of arrows pointing to A

**Definition:**  *A collection of arrows from object X to object Y in category Q is called a hom set and written as homQ (X, Y). A hom functor*  *maps an object X in category Q into a set, which is an object in category Set. A hom functor also maps an arrow f in category Q into a function, which is an arrow in category Set. A function maps a set to another set in category Set (see*  Fig. 6a  *for hom sets and 6b and c for hom functors).*

Among hom functors, one that is obtained by fixing an object A in category Q is called hom functor hA. Let us go back to our familiar example: an object A for Apple. Then, hom functor hA can be understood as “all relationships that Apple has with other objects in category Q.” Formally, hA is a functor from category Q to category Set. This is because hA maps objects Berry (B) and Cherry (C) in category Q into homQ (A, B) and homQ (A, C) in category Set, which can be understood as “how Apple relates to Berry in all possible ways” and “how Apple relates to Cherry in all possible ways.” hA also maps an arrow f to homQ(A, f), which is an arrow in category Set. Note that an arrow in Set is a function from a set to another set. For unfamiliar readers, homQ(A, f) is a little confusing. To understand this, recall that f is one of the arrows from object A to B in category Q, which can mean “nearly indistinguishable,” for example. Let us single out one of the arrows in homQ (A, B) as an arrow “a,” which means A is “nearly indistinguishable in its color” with B. Then, a can be composed with f to obtain a;f. This compositional situation means as follows: A is related to C (in a sense of “a;f”), where C is related to B (in a sense of “f”), which is related to A as “nearly indistinguishable in its color”. Note that, in categories where there are more than one arrows between objects, an arrow from A to C, which means “nearly indistinguishable in its color,” may be a distinct arrow from a;f. hA(f) maps a particular arrow from A to B into the corresponding arrow from A to C. If you consider arrows as elements in a set, this means that hA(f) is a function between two sets of arrows, that is, homQ (A, B) and homQ (A, C).

Note that until the last paragraph, we have always considered only one arrow from one object to the other, that is, we have simply considered categories of preorder in order to simplify various concepts in category theory. However, we will need to consider many arrows from one object to another object. homQ (A, B) is a powerful conceptual tool to think about such situations. We can consider homQ (A, B) as a list of properties of Berry in terms of relationship from Apple.

Pushing this idea further, we can denote a list of properties of all objects in category Q from the perspective of Apple as homQ (A, -) = hA. We use “-” to denote the argument in which any object in category Q can be substituted. For example, homQ (A,-) contains homQ(A, B) (i.e. a set of arrows from A to B, listing the properties of Berry from Apple’s viewpoint) as well as homQ(A, C) (i.e. a set of arrows from A to C). Thus, hA can be interpreted as all sets of arrows emanating from A to characterize the viewpoint of Apple. The dual notion is homQ (-, A) = Ah, which can be interpreted as all sets of arrows pointing to Apple. In other words, Ah is a list of properties of Apple, from the viewpoints of each and every object in category Q. These concepts are schematically represented in Fig. 6d. hA and Ah are likely to be useful to consider the issue of subjective viewpoints in consciousness research.

### What can we say from the Yoneda lemma?

The Yoneda lemma says:

*For each object A of C, the natural transformations Nat (hA, F) ≡ Hom(Hom(A,-), F) from hA to F are in one-to-one correspondence with the elements of F(A), that is, Hom(Hom(A,-)F) **≅** F(A). Moreover, this isomorphism is natural in A and F when both sides are regarded as functors from C × Fun(C, Set) to Set.*

Due to the technicality, we will omit the proof of the Yoneda lemma here. The readers who grasped all concepts introduced so far can understand the proof in a standard textbook of category theory (Awodey 2010; Leinster 2014). The important theorem that follows from the Yoneda lemma is as follows:

**Theorem: For objects A and B in category Q, hA** **≅** **hB is a necessary and sufficient condition for A ≅ B.**

Here, A **≅** B means that A and B are isomorphic objects in category Q. hA **≅** hB means that there exists a natural equivalence between hA and hB. A natural equivalence is a natural transformation from functor hA to hB, which is invertible. Remember a functor category, which we introduced previously. Given that hA and hB are both functors from category Q to category Set, they are objects of Fun(Q, Set). Thus, “hA ≅ hB” means hA and hB are isomorphic objects in Fun(Q, Set). In other words, there is an invertible natural transformation (i.e. a natural equivalence) between them.

Let us translate the theorem into English. hA (and hB) means all relationships that A has with all other objects in category Q. Thus, hA ≅ hB means that all relationships for A and those for B are “naturally convertible” to each other (note that hA and hB includes the relationship from A to B and B to A as well). If A and B are isomorphic objects in category Q, then it is relatively easy to prove hA ≅ hB. However, a nontrivial mathematical fact is that hA ≅ hB can prove A ≅ B. Even if it is impossible to directly compare A and B, we can make a conclusion about it by examining how A and B relate to others. This approach is akin to the approaches taken in other fields; when studying meaning in semantics, environments in ecology, and astronomical objects in cosmology. Of course, the exact and precise approach is taken in mathematics. Here, we are proposing its application for consciousness research.

Some readers may think that hA ≅ hB is more difficult to test empirically than A ≅ B. Surprisingly, however, there are many cases where it is overwhelmingly easier to check hA ≅ hB than A ≅ B. There is no shortage of examples in mathematics. Next, we consider it in the context of consciousness research.

### A simple application of the Yoneda lemma in consciousness research

**Figure 7.**
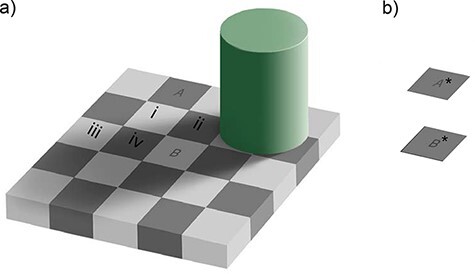
Applying the Yoneda lemma to category Q. (a) Checkershadow Illusion. The two squares labeled A and B look “different” in brightness as in complementary squares in a checkerboard. (b) Removing the surrounding of A and B to obtain A* and B*, whose brightness looks the “same”

Let us apply the above theorem for category of contents Q. To gain some insights, we consider the Checkershadow illusion (Adelson 1995) (Fig. 7a). In this “illusion,” square A looks very dark, while square B looks quite bright. In Fig. 7b, we show square A* and B* without any background, which makes it easy to see that the brightness of A* and B* are physically the same. The striking effect of this “illusion” does not reduce even if you try to cognitively convince yourself that it is just a 2D image or that the direction of the shadow in the image is contradictory to the lighting condition in your room. It exemplifies the fact that the subjective experience of brightness of an object strongly depends on the context of the objects.

Are the brightness of A and B the same? Can we define the subjective brightness of A and B? If there are individual differences in how we perceive brightness, is there any way to systematically examine and characterize subjective brightness?

Let us consider squares A and B in Fig. 7a from a perspective of category theory. Within category Q, A and B are not isomorphic. Why? Here, we consider objects as squares denoted by A, B, i, ii, iii, and iv and arrows as “nearly indistinguishable” as before.

First, consider if there is an arrow from A to i. Due to the clear boundary between A and i, they look quite different; thus, there is no arrow. Meanwhile, there is an arrow from B to i partly due to the fact that the bottom right part of i is indeed the same as that of B. However, if you pay attention to the top left part of the i, it becomes clear that i has a gradation and that i and B are not that similar. Yet, the cylinder on the top right provides an explanation of this difference within i as an effect of shadow. Furthermore, the overall configuration of the checkerboard also facilitates the sameness between B and i. In the end, we conclude the existence of an arrow from B to i. At this point, there is already a difference between a collection of relationships between A and the other objects and those between B and the others; thus, A and B cannot be isomorphic. This prediction coincides with our subjective phenomenology of “difference” between A and B, which provides a preliminary support of our framework of category Q.

What has been said above applies similarly to i, ii, iii, and iv. According to our framework, this collection of arrows created a conscious experience where A and B do not look the same. Meanwhile, the theorem predicts that A and B should look the same if we erase the sources that generate the discordances between arrows from A and arrows from B. And this is a more meaningful aspect of the theorem: if hA ≅ hB, then A ≅ B. The sources of discordance may include the green column and its shadow, the regularity of the checkerboard pattern, etc. Let us consider any points in the display as a potential object. Here, we assume A has a “nearly indistinguishable” arrow with a set of objects. Note that this set of objects include A itself (as “identity”) and B. If B has the arrow with the same set of objects, then mathematically, from hA ≅ hB, we can prove A ≅ B.

In fact, after painting squares i, ii, iii, and iv into a white color as in the background, one of the authors (H.S.) now feels that A and B look the same. The other author (N.T.) feels that A and B became much more similar than before but they are still distinguishable. By removing further contextual cues, as in Fig. 7b, A* and B* look the same to most people. At this point, some readers may find that the arrows that we have adopted so far, signifying “nearly indistinguishable,” are too coarse to be useful in this situation. To capture different degrees of perceived similarity, we believe that “enriched category” will be useful (Lawvere 1973; Leinster and Meckes 2017; Fong and Spivak 2019; Tsuchiya *et al.* 2021). The Yoneda lemma is also known to exist in the enriched category (Bonsangue *et al.* 1998); thus, our argument here still holds in the enriched category.

Note that we are not claiming that this is a novel explanation about the Checker shadow illusion. In fact, the application of the Yoneda lemma is never surprising as long as we work on preorder sets, where there exists at most one arrow between objects. Unfortunately, there have been very few empirical psychophysics studies that examined multiple arrows between objects so far. This is a highly promising avenue of future research.

In sum, the Yoneda lemma predicts that two objects A and B should look the same if we eliminate the discordances of relationships between A and the others vs B and the others.

## Discussion

In this paper, we introduced categories of consciousness in several forms and showed that the Yoneda lemma can be applied to obtain novel perspectives and predictions on consciousness. While the exact and concise definition of consciousness remains difficult, its characterization through indirect characterization of its relationships to others using convergent methods (see, for example, (Velmans 2009)) is actually a valid way as guaranteed by category theory and, in particular, the Yoneda lemma.

We can already use some tools from category theory to characterize some aspects of consciousness in terms of level and contents in relational terms, as we demonstrate with some simple toy models. As the research makes progress, we surmise that we can possibly characterize all aspects of consciousness in relational terms in principle. For other related proposals of a relational characterization of concepts, see Chalmers (1996), Edelman (1996), Goldstone *et al.* (2005), Kleiner (2020), Loorits (2014), Signorelli *et al.* (2021), and Fink *et al.* (2021).

As a future prospect, we think it is critical to consider various categories of consciousness within a larger structure of category of consciousness categories. Categorical equivalence between two conscious contents categories clarifies “in what sense” these consciousnesses are essentially the same.

The importance and novelty of our proposal here can be better appreciated by the following criticism on similarity judgment in consciousness research by Pautz (2019):

“To see how Similarity-Congruence falls short, let us return to hypothetical case … some unfamiliar sentient organism, Karl. You want to determine exactly what experiences Karl has… Karl is presented with three objects consecutively … three T-shapes such that T1 is more like T2 than T3. Then, given Similarity-Congruence, you can deduce that Karl has some trio of experiences, E1, E2, and E3, such that E1 is more like E2 than E3. But, as a simple point of logic, Similarity-Congruence is not logically strong enough to tell us precisely what those experiences are… it doesn’t tell us whether they are colour experiences of similar shades of red, or whether they are colour experiences of similar shades of green. In fact, it doesn’t tell us whether they are experiences of colour or experiences of (say) smell. That is, it doesn’t entail the specific, determinate qualitative contents of those experiences.”

Our proposal, specifically with the Yoneda lemma, is all about the power of “a collection of arrows (or relationships)” that is necessary to determine the color experience of red or green, the smell of fish, the sound of a bell, etc. And the essential role of a collection of relations does make sense in light of the fact that there has been no clinical report of a patient who lost color of red but not others. When brain damage causes color blindness, the reported cases are all about the entire loss of color experience (and associated concepts) in specific visual location (Gallant *et al.* 2000) but not a particular color or range of colors.^2^ Similar things can be said about visual motion (Zihl *et al.* 1983). Loss of a single category of perception, such as faces (e.g. prosopagnosia) and objects, has been linked to some specific brain lesions (Milner and Goodale 1995; Kanwisher and Yovel 2006) but rarely on a particular face [except for the loss of “familiar” faces and objects, called “Capgras Syndrome” (Ramachandran and Blakeslee 1998)]. On the other hand, loss of a single linguistic concept (i.e. forgetting) is common. Why can we lose a specific single concept in the linguistic case but not for the experiences of color or motion, which is bound to the retinal locations? While anatomical localizations and functional mapping in neuroscience can give us a hint, they do not address this theoretical question.

We believe that one possible answer to this riddle is to do with the Yoneda lemma. We hypothesize that the essence of the color experiences resides in relations with other colors (which may or may not be perceived at a given time), that is, without the entire sets of relations, color experiences just cannot emerge. On the other hand, we hypothesize that the essence of linguistic conceptual experiences [if any (Kemmerer 2015; McClelland and Bayne 2016)] is its relational structure that allows one concept to be missing from the web of relations. In retrospect, therefore, the essential role of a collection of relations in consciousness may be obvious (Palmer 1999). Yet, we are not aware if a collection of massive similarity ratings has been ever collected or considered in consciousness research. Replacing the characterization of conscious experiences with a collection of comparative descriptions with other experiences can have a mathematically solid foundation: the Yoneda lemma in category theory.

Combining the perspective of the Yoneda lemma with quantitative theories of consciousness, such as IIT (Oizumi *et al.* 2014; Tononi *et al.* 2016), we should be able to establish a new research program into animal consciousness (Tsuchiya 2017). Pioneering works in monkeys have introduced highly creative behavioral experimental tasks, such as binocular rivalry (Leopold *et al.* 2003) and no-report paradigms (Wilke *et al.* 2009), well beyond simple button press reports, which poorly characterize consciousness even in humans [also see Matsuzawa (1985) and Matsuno *et al.* (2004)]. These animal researchers have already brought various “arrows” in category Q for nonhuman primates. This type of research can be extended to other animals, such as dolphins, birds, octopuses, mice, and even insects such as flies (Boly *et al.* 2013; Barron and Klein 2016, Leung *et al.* 2021).

What other new perspectives can category theoretical analyses bring into consciousness research? Normally, researchers consider properties of the external world, such as the amount of light, as objective and physical reality. And, we call our perception “veridical” under the situation, where our subjective experience matches with these objective properties as in Fig. 7b. On the other hand, we call our perception “illusory” when our perception disagrees with the objective properties as in Fig. 7a. This “disagreement” invites some to call this figure as Checkershadow “Illusion.”

However, the very usage of the term “illusion” already implies the world view of the researchers. To some, what is “real” is the outside world and what we perceive can be “illusory.” This view is not necessarily endorsed by many consciousness researchers, especially by those who take phenomenology more seriously [also, see an argument from evolutionary game theory to claim that conscious percepts should not be “veridical” in this sense (Prakash *et al.* 2020)].

Category theoretical perspective allows researchers to keep a distance from metaphysical debates and to focus on what is empirically possible to investigate about conscious experience: its relationships with other experiences. Such an attitude is ontologically neutral. This, in turn, can potentially facilitate an interdisciplinary investigation of the contextual effects across experimental psychologists, neurophysiologists, and computational neuroscientists, philosophers, mathematicians under the same hood. To accelerate collaborations with category theory and some mathematical theories of consciousness, such as IIT, it will be necessary to develop further notions in stochastic categories (Manin and Marcolli 2020), information structures (Baudot and Bennequin 2015; Baudot *et al.* 2019), enriched categories (Tsuchiya *et al.* 2021), and category algebra (Saigo 2021).

Some readers may think that the Yoneda lemma application in Fig. 7 is an overkill as A and B can be directly compared in that example. Of course, this is a simple example to make a point. However, various controversies surrounding consciousness research are rooted in a question of whether conscious experiences are equivalent between two conditions, under which direct comparisons are difficult. For example, with or without paying attention, are conscious experiences essentially the same (Block 2007; Cohen *et al.* 2016; Haun *et al.* 2017)? Are the foveal vision and peripheral vision equivalent? What is, if any, the effect of expectation on conscious experience? In these situations, objects are difficult to compare in two conditions directly.

The indirect approach of the Yoneda lemma is especially effective in these situations. What is particularly powerful is that it makes an explicit prediction about our phenomenology. For example, if similarity relationships between object A and others are the same as object B and others, then A and B should be experienced as equivalent. And the prediction is possible to test empirically through experimentation. Category theory, indeed, is not just a conceptual framework to summarize what we already know. Its power originates from its predictions as we demonstrated in a preliminary way in Fig. 7. Researchers can make meaningful predictions about various outcomes in other situations where contextual effects play a key role, for example. Contextual modulatory effects are one of the essential features of conscious experiences in any modalities; thus, its applicability is likely to be vast.

## Conclusion

In this paper, we intentionally limited most of our examples into cases where up to one arrow can exist from one object to the other (preorder category). However, it is easy to extend our category Q to have multiple arrows between objects, such as “nearly indistinguishable in terms of X,” where X can be color, shape, size, location, etc. The richer the arrows, the better the structure of the category can be understood. In addition to what we introduced in this paper, there are many more tools in category theory, which are likely to be illuminating in consciousness research. Some of these conceptual tools will clarify complex theoretical concepts about consciousness, which have been discussed by philosophers and psychologists for long. Such conceptual clarification will inspire further theoretical and empirical research ideas to come in the near future.

## Footnotes

1 We assume that many readers of this article are unfamiliar with category theory and have no or little background training in mathematics. These readers are our primary target audience. To make the mathematical aspects as accessible as possible, we provided informal interpretations of mathematical concepts and their concrete examples in the context of consciousness research. We found this highly effective in introducing category theory to scientists with no or little mathematical background (Spivak 2014).

2 Note that most people with color deficiency do not report having loss of a particular red and/or green. In fact, previous studies suggest these color-deficient people may be experiencing unique color dimensions, as revealed by the similarity rating experiments (Bosten *et al.* 2005).
